# Noninvasive Self-Powered Iontophoresis Mask Based on a Water-Driven Fiber Battery

**DOI:** 10.34133/research.0667

**Published:** 2025-04-23

**Authors:** Yiwen Wang, Yalin Tang, Ming Li, Tong Xu, Xuyan Lu, Deteng Zhang, Ning Yu, Mingwei Tian

**Affiliations:** ^1^ Research Center of Health and Protective Smart Textiles, State Key Laboratory of Bio-Fibers and Eco-Textiles, College of Textiles and Clothing, Qingdao University, Qingdao 266071, China.; ^2^ Institute of Neuroregeneration and Neurorehabilitation, Qingdao University, Qingdao 266071, Shandong, China.; ^3^ Department of Anesthesiology, The Affiliated Hospital of Qingdao University, Qingdao, China.

## Abstract

Facial masks are often used to treat skin problems, and the introduction of microcurrent ion penetration technology can improve drug penetration and help facial tissue repair. However, most microcurrent stimulation masks contain a direct current power supply and require external power sources, resulting in inconvenient portability and use. Herein, we provide a noninvasive self-powered iontophoresis mask with a water-driven power supply, which is continuously prepared by self-constructing equipment to continually construct a zinc–manganese fiber battery (Zn-Mn@FB) and then seamlessly integrated with a nonwoven cellulose-based superabsorbent fiber substrate. The mask can be activated by water and is simple and portable to use. Zn-Mn@FB demonstrated a capacitance retention of 65.22% (1,000 cycles) and a specific discharge capacity of 27.33 mAh/g (10 cm), which improved with an increase in battery length to up to 41 mAh/g (30 cm). The iontophoresis mask exhibited a stable current within the safe range of 0.09 to 0.59 mA (within 800 s) after water activation, and the drug penetration area increased by 102.64%. The platform is expected to become a practical device for enhanced transdermal drug delivery in the medical field, with the potential to integrate additional components for expanded functionality and productization in the future.

## Introduction

Masks covering the skin are used to noninvasively solve facial skin problems, and small-drug molecules provide anti-skin acne, whitening, hydration, brightening, and other effects through transdermal penetration. Recently, ion penetration technology has gained much attention for the improvement of the drug penetration performance of facial masks. By applying a low-intensity electric field to the skin, the drug penetrates into the skin tissue in the form of ions, thereby improving the efficiency of wound healing, keratinocyte migration, muscle movement promotion, and skin elasticity restoration [1,2]. Under the stimulation of a microcurrent, small-molecular-weight (<500-Da) molecules can pass through the cuticle with higher efficiency [3], promote the production of more adenosine triphosphate, and enhance collagen density and fibroblast proliferation, thus reducing wrinkles [4,5] and increasing skin elasticity [1,4]. At present, there are already commercially available direct-current (dc)-powered ion electrophoresis beauty devices. However, in most microcurrent stimulation products, the assembly of the dc power supply and mask relies on a power cord or a traditional internal metal-based battery, which requires an external power supply, causing inconvenient portability and professional guidance to use. To solve these problems, the use of an internal power supply to replace the external power supply scheme can effectively improve the applicability of electrical dressing equipment.

Among existing internal power supply devices, the self-powered fiber battery combines a small size, softness, easy weaving, and a high safety factor, which makes it suitable for replacing an external power supply. Recently, a variety of self-powered fiber batteries with high energy storage power supply performance, excellent flexibility, and good biocompatibility have been widely reported, providing new opportunities for the energy supply of electrical dressing equipment [6–9]. High-performance wearable fiber-based batteries can be obtained by incorporating polymeric gel electrolytes into the aligned channels of fiber electrodes and the gaps between fibers [10,11]. They have a high power conversion efficiency (7.95%) and high durability under 5,000 deformation cycles (more than 90%), showing excellent application prospects in the field of flexible sensing and health protection. Moreover, in related reports of lightweight and flexible energy storage devices, zinc-ion batteries have become a research hotspot because of their advantages of low cost, high specific capacity, and high safety. The current research on zinc-ion batteries mostly focuses on performance improvement and material research and development, and the battery length is only about 10 cm [12]. Although the battery length can be increased using series batteries, there is a risk of loosening and leakage at the junction between batteries, which greatly limits practical applications. Therefore, more efforts are needed to develop a continuous and long-fiber battery for electrical dressing equipment.

Moreover, the safety performance of the electrolyte of a zinc-ion battery is also important. At present, the electrolytes of zinc-ion batteries mostly use polymers such as polyvinyl alcohol (PVA), gelatin, polyacrylamide, polyvinyl oxide, and polyacrylonitrile [13–16]. Polymer gel electrolytes have excellent mechanical properties, flexibility, good adhesion to human skin, and biocompatibility, which makes them an ideal platform for a new generation of wearable sensors. However, polymer gel electrolytes have poor water retention, and the battery fails after the water evaporates and cannot be reused. It is worth mentioning that the strategy of integrating cellulose-based superabsorbent fibers (SAFs) with traditional nonwoven fabrics (NWFs) is proposed to overcome the above problem. The enriched nanopore network in a nonwoven fiber and the frame structure on the fiber surface can form interconnected remote ion transfer pathways inside and outside the fiber, thus providing a pathway for ion transport [17,18]. In contrast to the above gel electrolytes doped with complex chemicals [19], cellulose and SAFs are considered to be safe and nontoxic and have good water retention and are proven to be able to produce disposable hygiene products, providing an ideal choice for internal power sources in electrical dressing equipment [20–22]. In addition, the human face has complex surface characteristics, including a large area, a complex curvature, multiple depressions, and protrusions in the eyes, nose, and mouth. Compared with the skin on the back, abdomen, and legs of the body, the facial skin shows relatively complex features [23]. Because of the particularity of the physical form and size of the fiber, SAFs have a fast water absorption speed, a soft feel, easy blending and processing, and easy material shaping without migration, which can meet the needs of facial skin fitting [24].

In this work, we propose a self-powered microcurrent iontophoresis mask using a zinc–manganese fiber battery (Zn-Mn@FB), which can be easily cut into desirable shapes. Zn-Mn@FB was constructed using self-built continuous equipment at room temperature to prepare long-fiber electrodes, and a mask was seamlessly integrated with superabsorbent NWF containing SAF interlayers to increase transdermal drug delivery. NWF absorbs water quickly, and the hydrogelatinization of the interlayer SAFs enables it to act as a battery electrolyte and recover when water evaporates, making Zn-Mn@FB water activable and recyclable. Zn-Mn@FB had a high specific capacity of 27.33 mAh/g when the current density was 20 mA/g, and with an increase in Zn–Mn battery length, the specific capacity increased to 41 mAh/g and then remained unchanged. Additionally, Zn-Mn@FB had stable cycling stability of 65.22% after 1,000 cycles. After activation, the iontophoresis mask maintained the current within the safe range of 0.09 to 0.59 mA for 800 s and the drug penetration area increased by 102.64%. The platform has good application potential in facial care.

## Results and Discussion

**Fig. 1.**
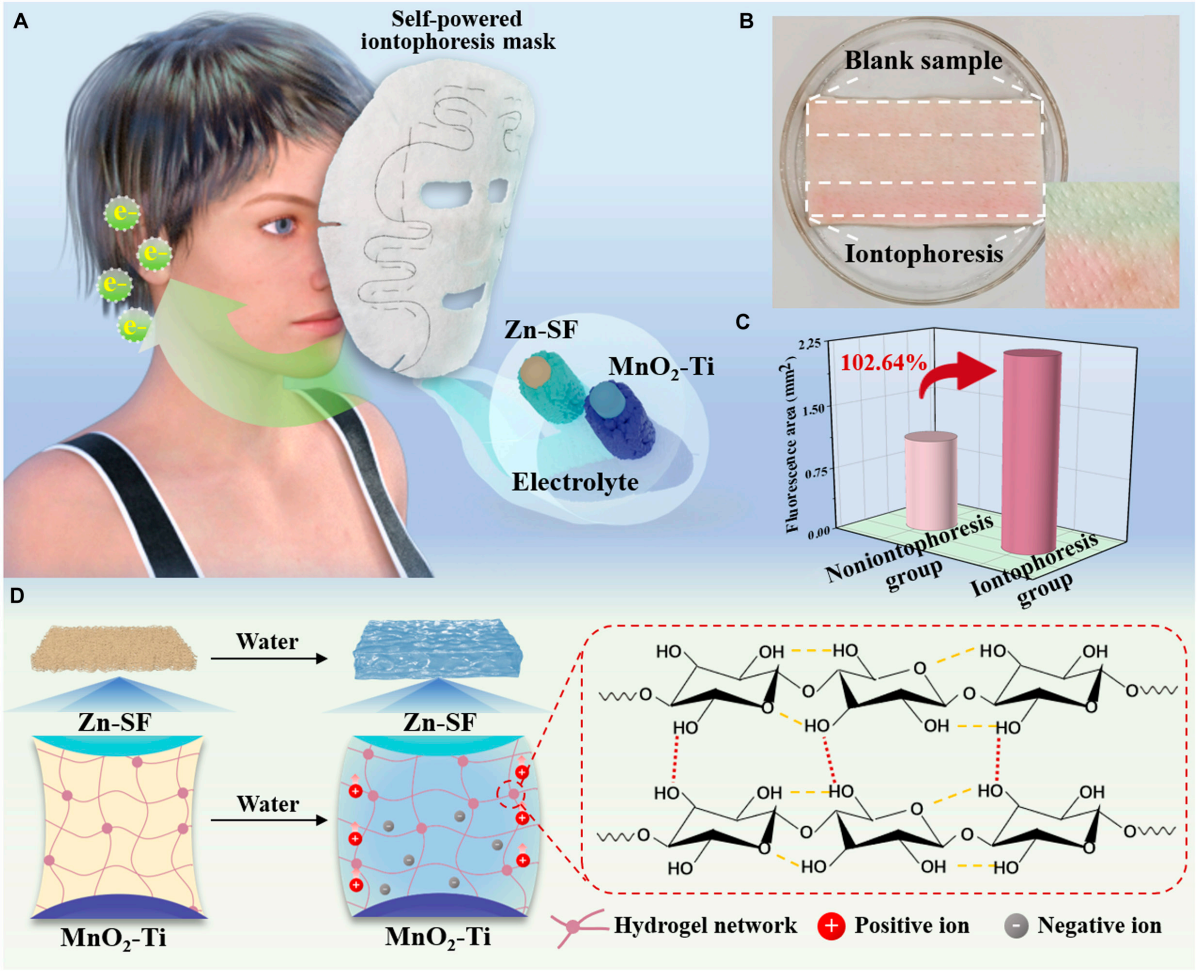
Structural design and mechanism of the self-powered iontophoresis mask. (A) Schematic illustration of the self-powered iontophoresis mask. (B) The effect of the mask on promoting drug penetration. (C) Comparison of drug penetration areas before and after mask application. (D) Gelation process of superabsorbent fibers (SAFs). Zn-SF, zinc-modified silver fiber anode; MnO_2_-Ti, manganese dioxide-modified titanium wire cathode.

The structural design and mechanism of the self-powered iontophoresis mask are schematically illustrated in Fig. 1. The whole mask was sewn using several Zn-Mn@FB units. Zn-Mn@FB was activated by water and consisted of a zinc-modified silver fiber anode (Zn-SF) and a manganese dioxide-modified titanium wire cathode (MnO_2_-Ti). Because of the small ionic radius, ion intercalation and deintercalation of Zn^2+^ could occur in the compound with a tunnel/layered structure [25]. Zn^2+^ was released from the negative electrode of the Zn-Mn@FB battery during discharge, which diffused further into the MnO_2_-Ti positive electrode. Then, it was removed from the tunnel of the MnO_2_-Ti positive electrode during charging and finally produced a reflux in the electrical circuit. The continuous low-intensity current delivered by the electrical circuit promoted the introduction of the drug into the skin. There was a clear color boundary between the surface of the iontophoresis sample and the blank sample (Fig. 1B), and the drug delivery efficiency was substantially improved (Fig. 1C). In addition, the current transport effect of Zn-Mn@FB was inseparable from the electrolyte. NWF had a gelling state when encountering water (Fig. S1). The gelation mechanism is illustrated in Fig. 1D. The structure of SAFs consisted of a linear chain of cyclic glucose molecules with a repeating unit of 2 dehydrated glucose rings bridged by C–O–C covalent bonds. Using SAFs as the intermediate layer of NWF, gelation was triggered by the core-suction effect. In the gelation process, SAFs could form a 3-dimensional network of gel fibers through the hydrogen bond interaction between nanofibers, which carried the flow of electrons and generated a conductive path.

**Fig. 2.**
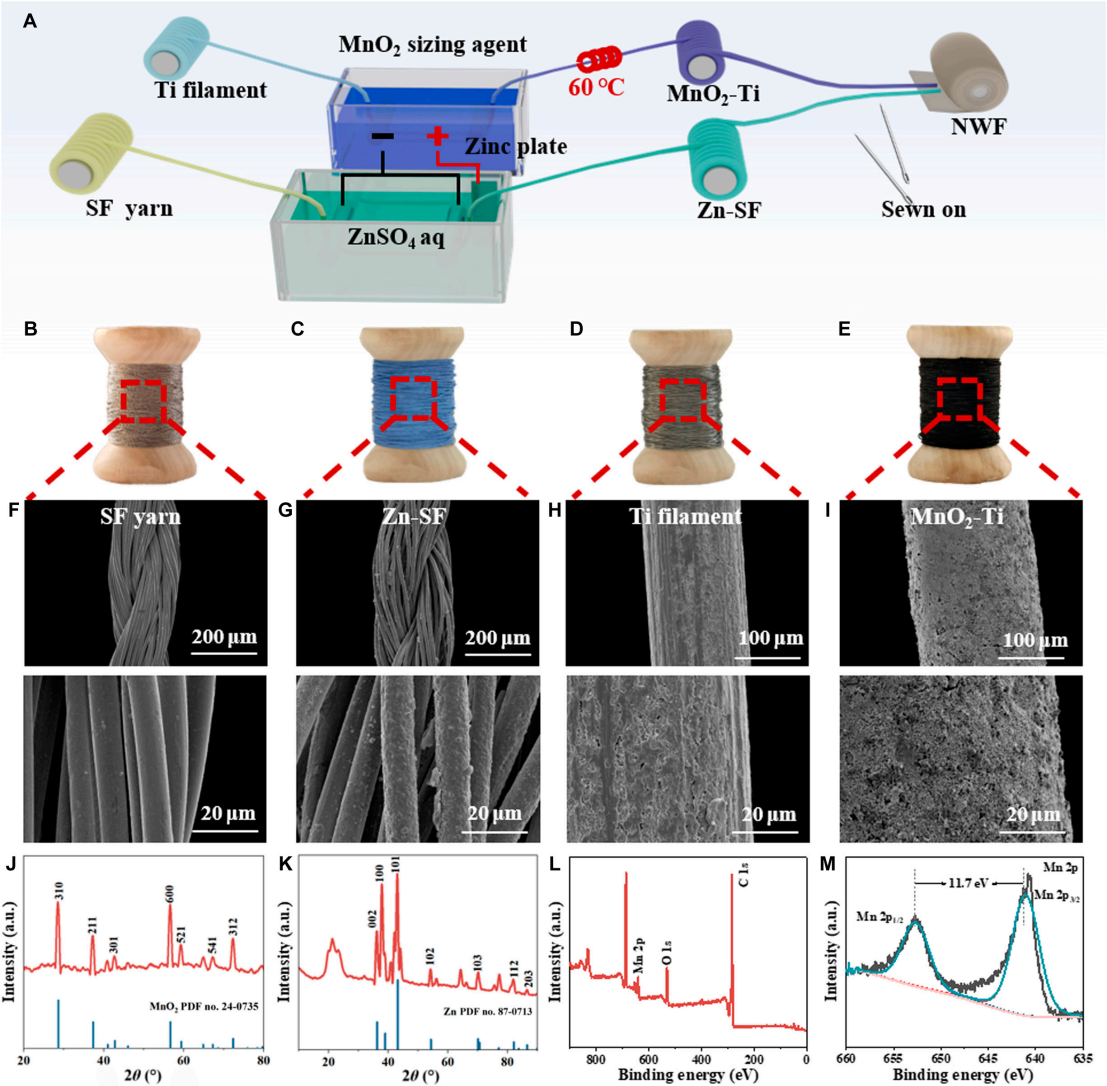
Microscopic structure and chemical structure of the electrodes. (A) Schematic diagram of the continuous preparation of the Zn-SF electrode and MnO_2_-Ti electrode. (B to E) Photographs and (F to I) scanning electron microscopy (SEM) images of silver fiber (SF), the Zn-SF electrode, a Ti filament, and the MnO_2_-Ti electrode. X-ray diffraction (XRD) patterns of the (J) MnO_2_-Ti electrode and (K) Zn-SF electrode. XPS (L) full spectrum and (M) Mn 2p spectrum of the MnO_2_-Ti electrode. NWF, nonwoven fabric.

Compared with paper [26,27], film [28–30], plastic, etc., textile fiber materials have excellent flexibility and strength, and their unique textile structure and flexible designability also enable them to be seamlessly combined with textile products to make wearable electronic products through textile processing technology [31–33]. The fiber structure of the zinc-ion battery gave it higher flexibility, and it was easier to integrate with the fabric through the weaving process, which was widely used in smart clothing. However, existing studies on flexible zinc-ion batteries mainly focused on electrode material modification and electrochemical performance improvement, and their application was limited to a safe and stable environment. There was a lack of research on fiber batteries under mechanical external forces (bending, torsion, heavy pressure, etc.). Most of the research content was also focused on the length of a few centimeters or more than 10 cm, although relatively large progress had been made in performance, but the length of the energy storage device greatly limited the design of the integrated process. Here, we continuously prepared Zn-SF and MnO_2_-Ti electrodes using self-constructing deposition equipment (Fig. 2A). In the process of electrodeposition, the influence of different electroplating voltages on zinc electrodes was discussed. Using zinc sulfate (ZnSO_4_) as the electrolyte, different deposition voltages were set and the morphology of the zinc layer deposited on silver-coated yarn (SF) was observed using an ultra-depth-of-field video microscope (Fig. S2). The results showed that when the deposition voltage was 2 V, the zinc layer morphology presented the best deposition effect, and the zinc layer was dense and evenly wrapped to the yarn, and there was no loose zinc material on the surface.

The resistance of Zn-SF enlarged slowly with increasing length (Fig. S3), while the resistance decreased first and then enlarged with increasing deposition voltage (Fig. S4). When the deposition voltage was 2 V, the lowest resistance of Zn-SF was 10.36 Ω, which further proved that the optimal deposition voltage of Zn-SF was 2 V. Therefore, 2 V was used as the deposition voltage in subsequent experiments. The fiber raw materials before deposition were compared with the fiber electrodes after deposition, and the photos and SEM images of SF, the Zn-SF electrode, the Ti filament, and the MnO_2_-Ti electrode were obtained (Fig. 2). The original SF was silvery gray with a smooth surface (Fig. 2B and F). Along with the fine growth of zinc nanoparticles on the SF surface, the surface of the Zn-SF electrode appeared silver-white (Fig. 2C and G). As exhibited in Fig. 2D and H, the surface of the Ti filament was free of impurities, and the surface of the MnO_2_-Ti electrode was uniformly loaded with manganese dioxide slurry, and the color was black (Fig. 2E and I). The fine and uniform arrangement of manganese dioxide nanoparticles and zinc nanoparticles provided more active sites for the intercalation and deintercalation of zinc ions, which was beneficial to improving the electrochemical performance of zinc-ion batteries. The effective preparation of MnO_2_-Ti (Fig. 2J) and Zn-SF (Fig. 2K) were confirmed by x-ray diffraction patterns. The full spectrum of the MnO_2_-Ti electrode clearly presented the presence of the C, O, and Mn elements (Fig. 2L and M). Moreover, the energy-dispersive x-ray spectroscopy image of the distribution of elements on the electrode surface (Fig. S5) also confirmed that Zn and MnO_2_ active materials were successfully attached and evenly distributed on the fiber electrode.

**Fig. 3.**
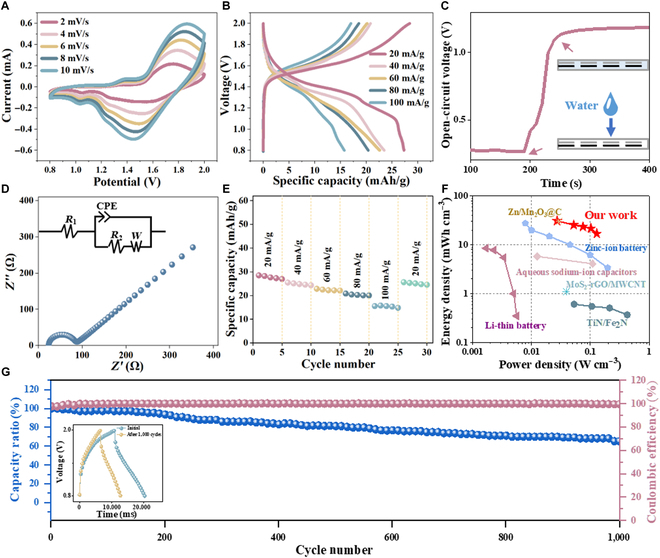
Electrochemical performance of Zn-Mn@FB. (A) Cyclic voltammetry (CV) curve at scanning rates of 2 to 10 mV/s. (B) Constant-current charge–discharge (GCD) curve at 20 to 100 mA/g current densities. (C) Open-circuit voltage response of Zn-Mn@FB. (D) Electrochemical impedance spectroscopy (EIS) spectrum of Zn-Mn@FB; the inset illustrates the equivalent circuit of the device. (E) Rate performance at different current densities. (F) Comparison with other works. (G) Cycle stability and coulombic efficiency of Zn-Mn@FB; the illustration shows the 1st and 1,000th charge and discharge curves. CPE, constant phase element; rGO, reduced graphene oxide; MWCNT, multiwall carbon nanotube.

Zn-SF and MnO_2_-Ti flexible fiber electrodes were seamlessly integrated into NWF to obtain zinc–manganese Zn-Mn@FB batteries. The redox peaks during Zn^2+^ dissociation and embedding of Zn-Mn@FB were observed at a scanning rate of 2 to 10 mV/s (Fig. 3A). There was no obvious deformation of the curve at different scanning rates, which indicated that Zn-Mn@FB could withstand fast charging and discharging. According to the charge–discharge curve in Fig. 3B, Zn-Mn@FB presented a good energy storage performance with a specific discharge capacity of 27.33 mAh/g when the current density was 20 mA/g. To verify the activation effect of the electrolyte on Zn-Mn@FB, the open-circuit voltage of Zn-Mn@FB with a length of 10 cm was measured. When 260 μl/cm^2^ of deionized water was added to the NWF, the open-circuit voltage increased substantially and a stable platform was obtained (Fig. 3C). A low charge transfer resistance reduced the transfer resistance of electrons and ions during the electrochemical reaction. The electrochemical impedance spectroscopy spectrum in Fig. 3D exhibited that Zn-Mn@FB obtained a low equivalent series resistance of 23.4 Ω and a charge transfer resistance of 65.3 Ω. The working stability of Zn-Mn@FB was further explored by observing the specific discharge capacity, as shown in Fig. 3E, when the current density was 20, 40, 60, 80, and 100 mA/g. The corresponding specific discharge capacities were 28.52, 25.47, 22.83, 20.83, and 15.56 mAh/g, respectively. In addition, after the above current density tests, the specific discharge capacities of Zn-Mn@FB under a 20 mA/g current density still reached 25.64 mAh/g. This was probably determined by the multidimensional nanostructure of the active material as well as the stability of the electrolyte. According to Eqs. 2 and 3, our Zn-Mn@FB achieved a maximum energy density of 37.16 mWh/g (30.94 mWh/cm^3^) at a power density of 0.0272 W/g (0.0226 W/cm^3^). A maximum power density of 0.128 W/g (0.106 W/cm^3^) was achieved at an energy density of 20.3 mWh/g (16.9 mWh/cm^3^) (Fig. 3F and Fig. S6). The comparison of the volume energy and power density of Zn-Mn@FB with those of other high-performance energy storage devices clearly exhibited that our Zn-Mn@FB was substantially superior to previously reported devices such as nitride solid-state batteries [34], zinc batteries [35,36], lithium batteries [37], water–sodium-ion batteries [38], and hybrid batteries [39]. In addition, the cyclic stability of Zn-Mn@FB showed that the capacitance remained 65.22% after 1,000 cycles and the coulombic efficiency was about 100% (Fig. 3G and Fig. S7). Zn-Mn@FB possessed good cycle stability, which provided the possibility of its long-term practical application.

**Fig. 4.**
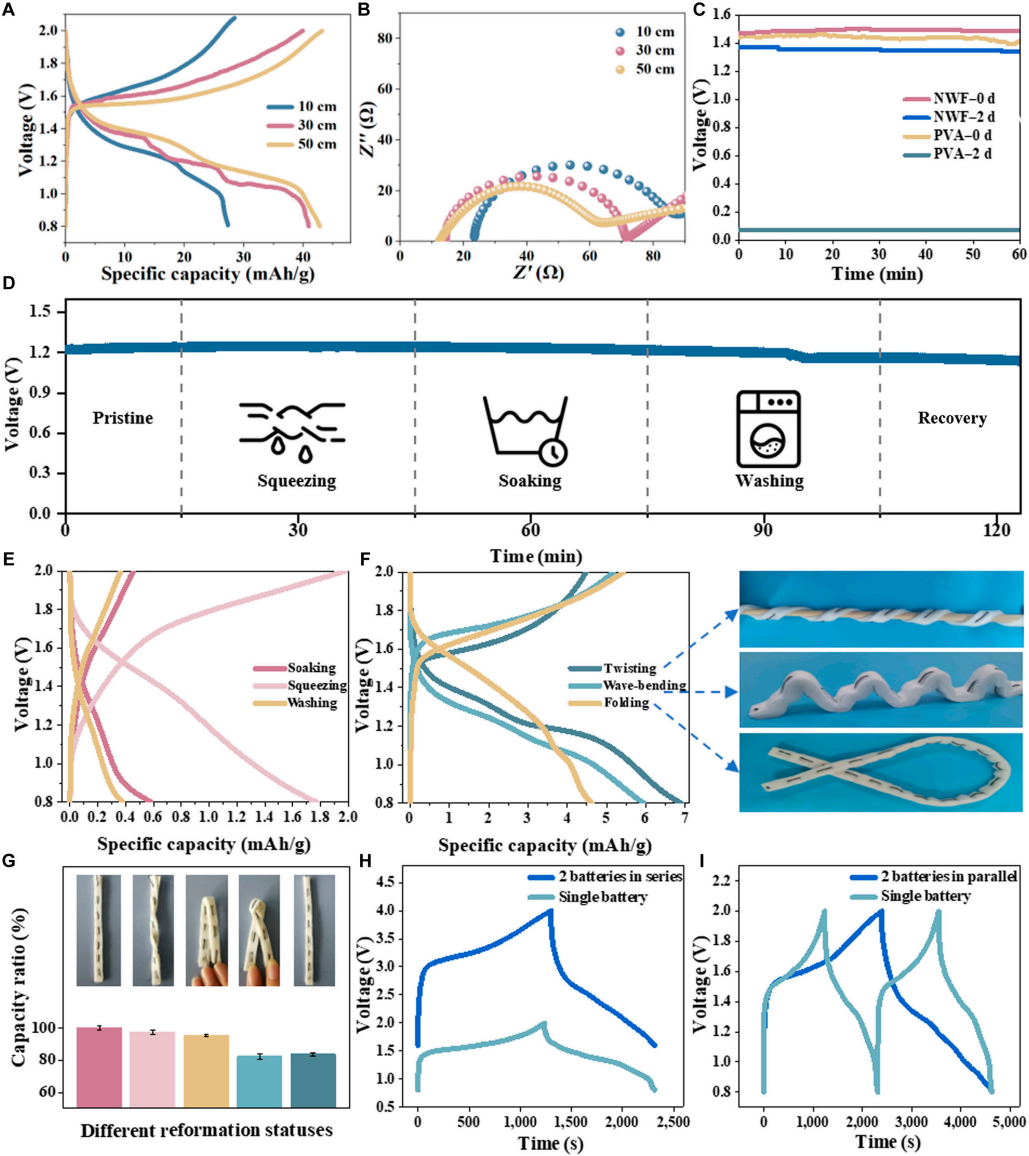
Wearable performance of Zn-Mn@FB. (A) Charge–discharge performance and (B) impedance at different lengths of Zn-Mn@FB. (C) Output voltage retention performance of different electrolytes. (D) Open-circuit voltage and (E) charge–discharge performance under washing conditions. (F) Charge–discharge performance and (G) capacitance retention under different deformations. (H) Series GCD curve and (I) parallel GCD curve of Zn-Mn@FB. PVA, polyvinyl alcohol.

The length of the energy storage device had a great effect on the design of the integration process, and an increase in length also affected the internal resistance of Zn-Mn@FB and the corresponding electrochemical performance. Flexible fiber batteries of different lengths were prepared, and analyses of their charge–discharge performance and impedance were carried out in order to investigate the effect of length on Zn-Mn@FB. The results showed that compared with a 27.33 mAh/g specific discharge capacity at a 10-cm length, the specific discharge capacity of Zn-Mn@FB increased to 41 mAh/g when the battery length increased to 30 cm, and that of Zn-Mn@FB increased to 40.6 mAh/g when the battery length increased to 50 cm (Fig. 4A). Moreover, the internal resistance of Zn-Mn@FB presented a decreasing trend with an increase in the length of the fiber battery (Fig. 4B). When the length of the fiber battery was 30 cm, the internal resistance was 14 Ω, and the internal resistance decreased to 12 Ω when the length was increased to 50 cm. PVA gel and NWF were used as electrolytes to observe the discharge to verify the durability of SAF electrolytes; as shown in Fig. 4C, the 2 kinds of gel electrolyte batteries have similar output potentials. Within 60 min, the output voltage of NWF was stable at about 1.17 V, while that of PVA gel was stable at about 1.15 V. However, after 2 d of standing, the output voltage of PVA gel was almost 0 V, and NWF still had an output voltage. This was mainly due to the complete loss of water in the PVA electrolyte, resulting in battery failure. Although the electrolyte of the fiber battery with NWF as the electrolyte also volatilized after 2 d of standing, the output voltage of the fiber battery was able to be maintained at about 1.15 V after water absorption again.

Zn-Mn@FB, as a wearable energy storage device, was supposed to meet the needs of daily wearability, including washable resistance, immersion resistance, and air permeability. The electrochemical performance of Zn-Mn@FB was tested under different mechanical deformation, soaking, and washing conditions. The mechanical deformation included squeezing, twisting, waveform bending, and folding. Zn-Mn@FB was mixed and washed for 10 min with water containing 1 wt% detergent, and dried at room temperature and activated with deionized water. As shown in Fig. 4D, under the conditions of squeezing, soaking, and washing, Zn-Mn@FB still provided a relatively stable open-circuit voltage under the above conditions. In addition, compared with that after squeezing, the specific capacitance of Zn-Mn@FB remained 32.92% and 21.32% after soaking and washing, respectively (Fig. 4E). After gradually complex mechanical deformations such as twisting, waveform bending, and folding, the discharge specific capacity loss of Zn-Mn@FB gradually increased (Fig. 4F), but it still maintained a high capacity retention rate. The capacity retention rates of Zn-Mn@FB after twisting, folding, and knotting were 97.48%, 95.14%, and 82.24%, respectively, and still reached 83.75% after the above various mechanical deformations (Fig. 4G). The above experiments displayed that Zn-Mn@FB maintained good flexibility and could adapt to the deformation required by daily wearing. In addition, to meet the power requirements for daily wearable use, a series–parallel connection to Zn-Mn@FB was used to achieve higher output voltage and current. The output voltage of 2 Zn-Mn@FB batteries in series was twice that of a single battery, and the working time was almost the same (Fig. 4H). Similarly, the output time of 2 parallel Zn-Mn@FB batteries was twice that of a single battery, and the operating voltage was the same (Fig. 4I), which fully proved that series–parallel connection could achieve the effect of expanding voltage and increasing current.

**Fig. 5.**
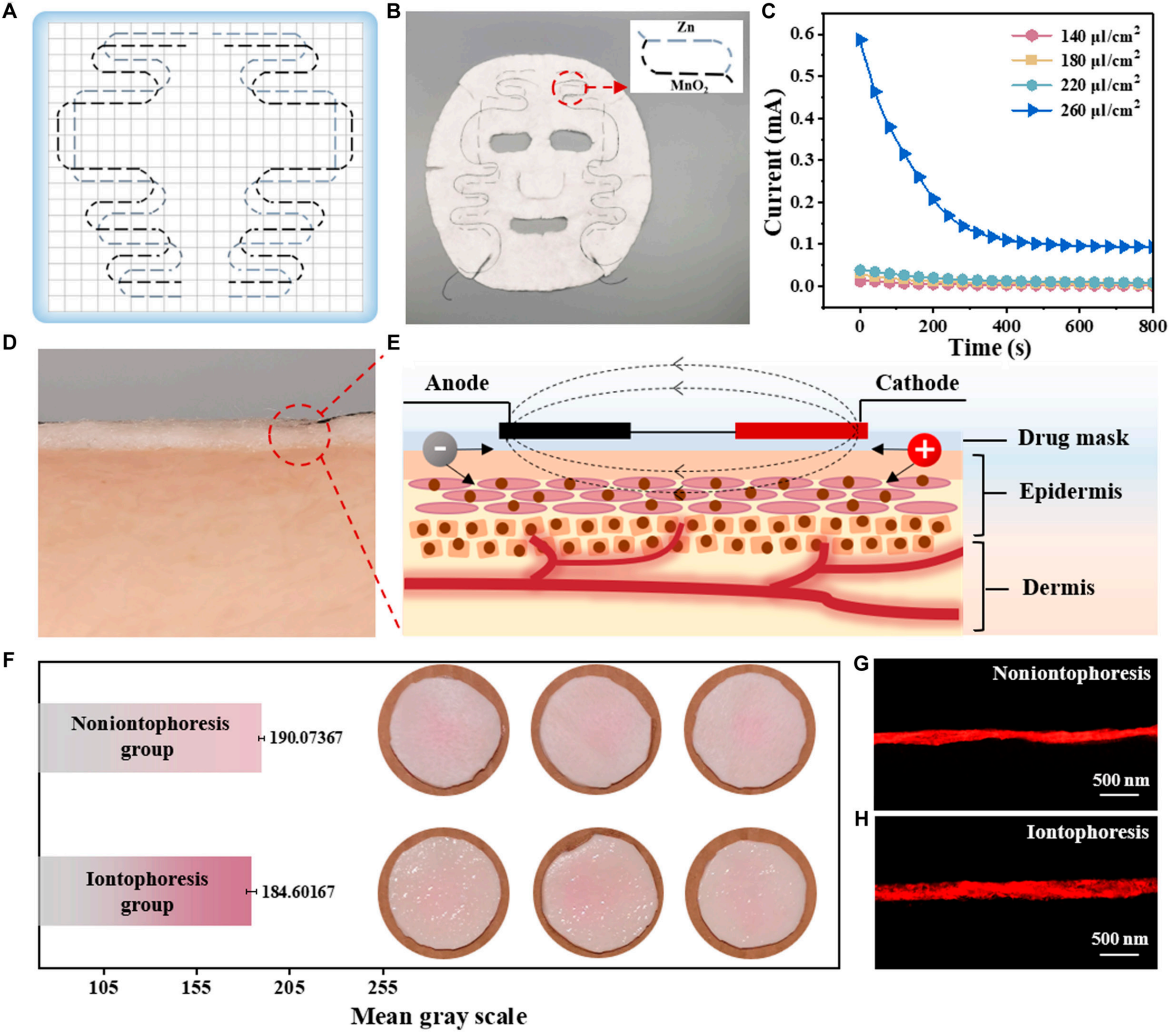
Application performance of the self-powered iontophoresis mask. (A) Embroidery design of the mask. (B) Physical picture of the mask; the small picture is that of a Zn-Mn@FB unit. (C) Output current images at different electrolyte volumes. (D) Application scenario diagram. (E) The microcurrent promotes the permeability mechanism. (F) Mean gray scale of the noniontophoresis group and the iontophoresis group, which was treated with iontophoresis mask for 1 h. SEM photos of the longitudinal section of pigskin in the (G) iontophoresis group and (H) noniontophoresis group.

Based on Zn-Mn@FB good energy storage, magnification performance, and wearable performance, we completed the design and production of the self-powered iontophoresis mask through a simple embroidery process. The graphic design of battery on the iontophoresis mask is exhibited in Fig. 5A. Zn-SF and MnO_2_-Ti fiber electrodes were arranged on the mask base cloth in regular shapes and were continuously sewn and interwoven to form several Zn-Mn@FB small units to form a closed loop. As shown in Fig. 5B, Zn-Mn@FB distributed at different locations on the face could generate a microcurrent when used to transport ions in the stratum corneum of the skin and raise the permeability of the cell membrane, thus increasing the amount and depth of transdermal drug delivery [23,40,41]. The microcurrent was able to promote the transdermal transport of small molecular nutrients such as hyaluronic acid. The data presented that currents less than 1,000 μA could promote adenosine triphosphate synthesis, and currents in the range of 100 to 500 μA could stimulate protein synthesis [42,43]. With reference to the average duration of the application of the mask, a current test of the mask with different electrolyte volume fractions was carried out within 800 s (Fig. 5C). When the NWF mask base cloth was not fully wetted, the mask provided a microcurrent of 0.038 mA or less, and after being fully wetted, the mask provided a microcurrent of up to 0.589 mA, which slowly decreased to 0.093 mA over time. In the soaked state, the current increase of the mask reached 0.964 mA, but the current decline curve was steeper (Fig. S8). This was probably due to the increase in ion exchange efficiency caused by electrolyte supersaturation and electrode contact short circuit, resulting in an increase in the discharge speed. The iontophoresis mask presented a gel state after exposure to water and was effectively fitted on human skin (Fig. 5D). The mechanism of the microcurrent promoting penetration is presented in Fig. 5E. Dropping water as an electrolyte activated Zn-Mn@FB to release a microcurrent from the mask, thus promoting the transdermal diffusion and penetration of drugs. For proof of concept in Fig. 5E, the process of applying a mask on fresh pigskin was simulated. The fluorescent dye rhodamine B was used to simulate the drug. It was observed that the fluorescence intensity of the skin surface of the iontophoresis group was higher than that of the noniontophoresis group, and the range was substantially expanded (Fig. 5F). The average fluorescence intensity of the iontophoresis group was higher than that of the noniontophoresis group as a whole by selecting an equal-diameter circle in the center of the sample for gray value analysis. Drugs on the skin without microcurrent stimulation mostly remained in the epidermis, and the penetration was uneven (Fig. 5G). The fluorescence band width of drugs on the skin stimulated by a microcurrent was larger and more uniform (Fig. 5H), and the drug penetration area increased by 102.64%, indicating that the iontophoresis group could effectively promote the penetration and absorption of drugs.

## Conclusion

In summary, Zn and MnO_2_ were loaded onto a substrate surface using self-constructing continuous chemical deposition equipment, and the obtained fiber electrode was seamlessly integrated with the superabsorbent NWF to prepare a noninvasive self-powered iontophoresis mask that enhanced transdermal drug delivery. The Zn-Mn@FB unit on the mask exhibited an ideal discharge specific capacity of 41 mAh/g (30 cm), a cycle stability of 65.22% (after 1,000 cycles), and an output current in the safe range of 0.09 to 0.59 mA (within 800 s), which effectively improved the drug delivery efficiency. Our work demonstrated that the prepared Zn-ion battery iontophoresis mask has a good application potential in enhancing transdermal drug delivery and is expected to provide a more practical microcurrent introduction device for medical cosmetology with the possibility of functional expansion and industrial production.

## Materials and Methods

### Materials

The silver fiber (SF) was purchased from Lutai Textile Co., LTD. The Ti filament was purchased from Kunshan Shengshijingxin Material Co., LTD. Anhydrous ethanol was purchased from Tianjin Guangfu Fine Chemical Research Institute. Zinc sulfate (ZnSO_4_) was purchased from Shanghai McLean Biochemical Technology Co., LTD. PVA was purchased from Sinopharm Group Chemical Reagent Co., LTD. Manganese dioxide (MnO_2_), *N*-methylpyrrolidone (95%), polyvinylidene fluoride (PVDF; 99%), and acetylene black (99%) were purchased from Qingdao Fengruixin Instrument Equipment Co., LTD. Rhodamine B was purchased from Qingdao Runhuan Science and Technology Engineering Co., LTD. Fresh pigskin was purchased from Hedong District Leisen Food trade. Superabsorbent nonwovens were purchased from Taian Youxin Materials Co., LTD.

### Preparation of the Zn-SF electrode

First, SF was immersed in anhydrous ethanol for 10 min by ultrasound, cleaned with deionized water, and then placed in the oven at 60 °C until completely dry. This was repeated 3 times to remove the wax and impurities on the surface of the silver-coated fiber. Then, ZnSO_4_ powder was dissolved in deionized water and prepared into 0.5 M ZnSO_4_ plating solution, which was fully stirred on a magnetic agitator until ZnSO_4_ was completely dissolved. The solution was poured into a plating pool, with the zinc plate as the positive electrode and SF as the negative electrode, and electrodeposition was carried out under the voltage applied by a regulated dc power supply. Finally, the zinc-modified fiber anode was collected by an automatic winding device with a winding speed of 4 r/s and named Zn-SF.

### Preparation of the MnO_2_-Ti electrode

PVDF was poured into a beaker containing an appropriate amount of *N*-methylpyrrolidone solution, thoroughly stirred on a magnetic mixer until it was completely dissolved, and then poured into MnO_2_ and conductive acetylene black in turn to obtain MnO_2_ slurry. The weight ratio of MnO_2_, conductive acetylene black, and PVDF was 7:2:1. Next, the surface of the titanium wire was coated with MnO_2_ slurry, dried by hot air at 60 °C. Finally, the MnO_2_-modified fiber cathode was collected by an automatic winding device with a winding speed of 4 r/s and named MnO_2_-Ti.

### Assembly of the iontophoresis mask

Zn-SF and MnO_2_-Ti were stitched into NWF with embroidery to obtain a Zn–Mn electrode unit as the test group, named Zn-Mn@FB. After that, several Zn-Mn@FB units were arranged on the mask base cloth according to the embroidery pattern in Fig. 5A to complete the assembly of the mask.

### Transdermal drug delivery experiment induced by the self-powered iontophoresis mask

The process of applying a mask on fresh pigskin was simulated. The observation group was divided into 2 groups, which were a blank sample and the iontophoresis mask driven by Zn-Mn@FB; 3 parallel samples were set for each group. After cleaning, fresh pigskin was cut into a circle of equal diameter and placed on a plate; 0.6 ml of 1 × 10^−3^ M rhodamine B was added to the center, and the sample size was 10 × 1 cm^2^. After treatment for 1 h, the fluorescence intensity between the cathode and anode on the surface of pigskin was observed. The surface samples were removed, and the pigskin was frozen in liquid nitrogen and sliced into 10-μm-thick specimens, which were then observed under a fluorescence microscope.

### Characterization

The physical morphology of Zn-SF electrodes under different deposition voltages was analyzed with a DVM6M ultradepth-of-field video microscope from Leica, Germany. The surface morphologies of SF, the Zn-SF electrode, the Ti filament, and the MnO_2_-Ti electrode were analyzed by scanning electron microscopy. The surface compositions of MnO_2_-Ti and Zn-SF electrodes were analyzed with a ESCALAB Xi+ x-ray diffraction photoelectron spectrometer from Thermo Fisher (Czech Republic). The crystal structure on the surface of the MnO_2_-Ti electrode was analyzed with a 3-kW SmartLab x-ray photoelectron spectroscopy instrument. The surface of pigskin was observed with a fluorescence electron microscope from Nikon Corporation.

### Electrochemical measurements

Constant-current charge–discharge, cyclic voltammetry, and electrochemical impedance spectroscopy tests were performed on Zn-Mn@FB with CHI660E and CHI760E electrochemical workstations made by Shanghai Chenhua Instrument Co., LTD. All tests were carried out at room temperature in the voltage range of 0.7 to 2.0 V. The volume specific capacitance ($C_{\mathrm{sp}}$), energy density (E), and power density (P) of Zn-Mn@FB were calculated from Eqs. 1, 2, and 3, respectively [44,45]:

$$C_{\mathrm{sp}}=\frac{\mathrm{It}\times 1,000}{V\times 3,600}$$ (1)

$$E=\frac{i\int U\left(t\right)\mathrm{dt}}{3,600}$$ (2)

$$P=\frac{E\times 3,600}{t}$$ (3)

where I is the discharge current, t is the discharge time, V is the volume, i is the current density, $U\left(t\right)$ is the voltage at time t, and dt is the time differential.

## Data Availability

All relevant data supporting the key findings of this study are available within the article and its Supplementary Materials file or from the corresponding authors upon reasonable request.
