# Supplementary material for: Noninvasive Self-Powered Iontophoresis Mask Based on a Water-Driven Fiber Battery
Source: Research (Wash D C). 2025 Apr 23;8:0667. doi: 10.34133/research.0667 (PMC12015099; doi:10.34133/research.0667)
Supplement: Supplementary 1 — Figs. S1 to S8 [file research.0667.f1.docx]

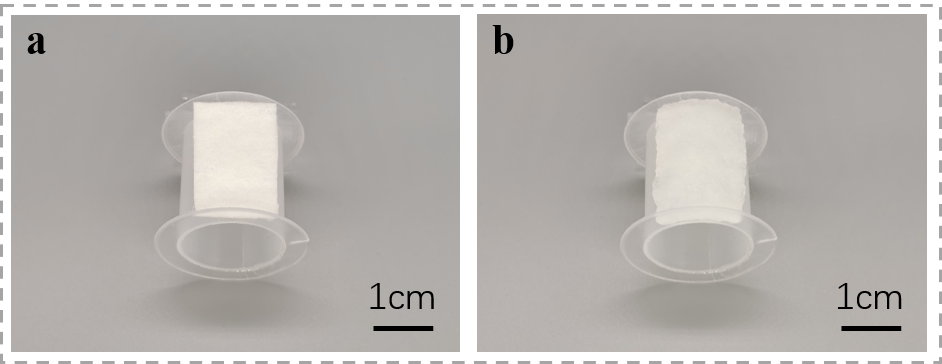


Fig. S1. NWF gelation diagram. (a) Dry state and (b) wet state of NWF.


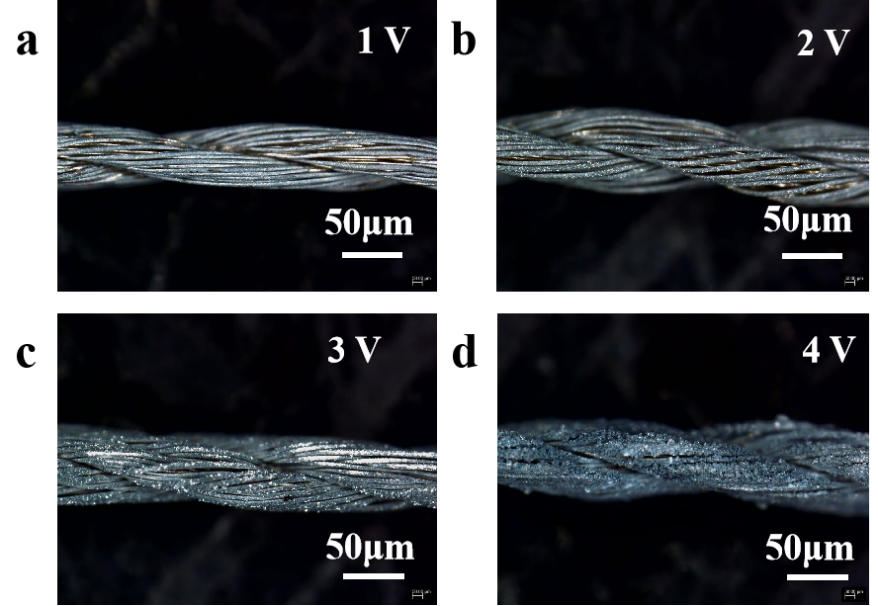


Fig. S2. Zinc electrode morphology at different deposition voltages of (a)1 V, (b) 2 V, (c) 3 V and (d) 4 V.


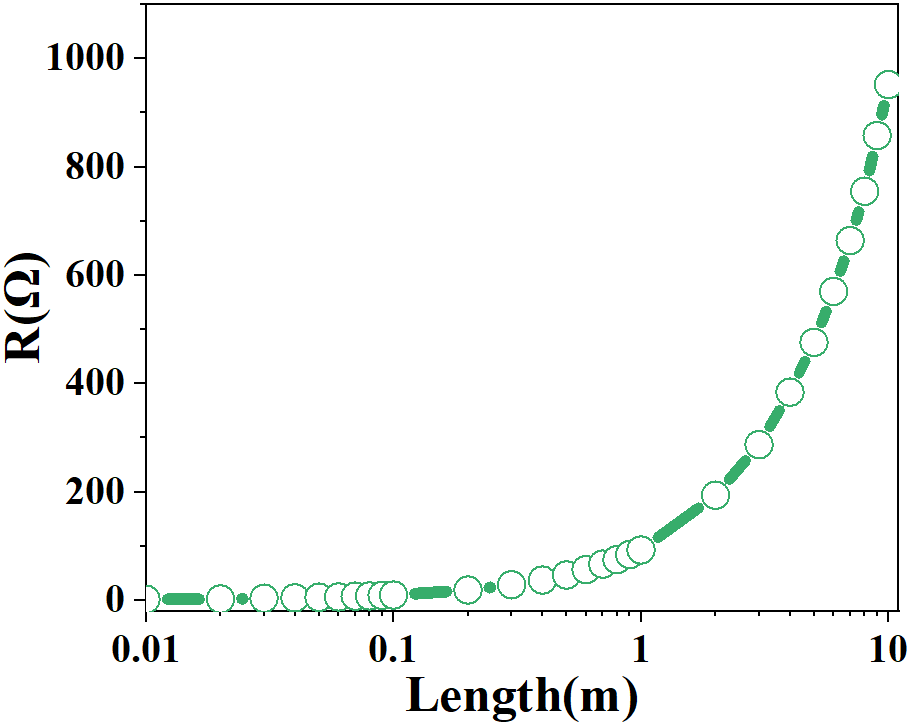


Fig. S3. Resistance of different lengths of galvanized yarn.


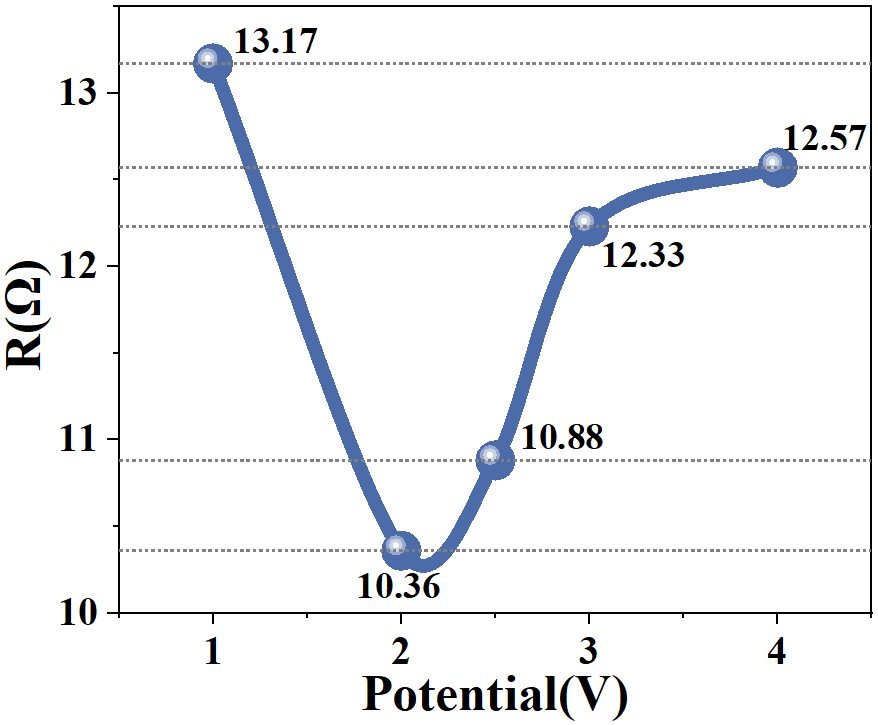


Fig. S4. Resistance of zinc electrode at different deposition voltages.


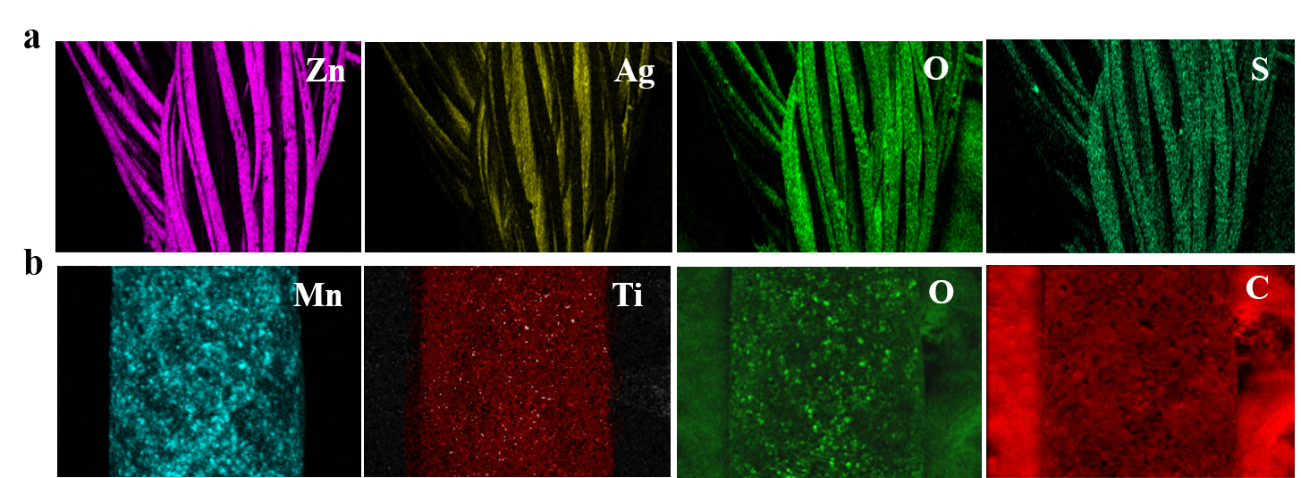


Fig. S5. EDS Mapping images. (1) Zn, Ag, O and S elements in continuous galvanized silver coated fiber electrode. (2) Mn, Ti, O and C elements in continuous manganese dioxide modified titanium wire electrode.


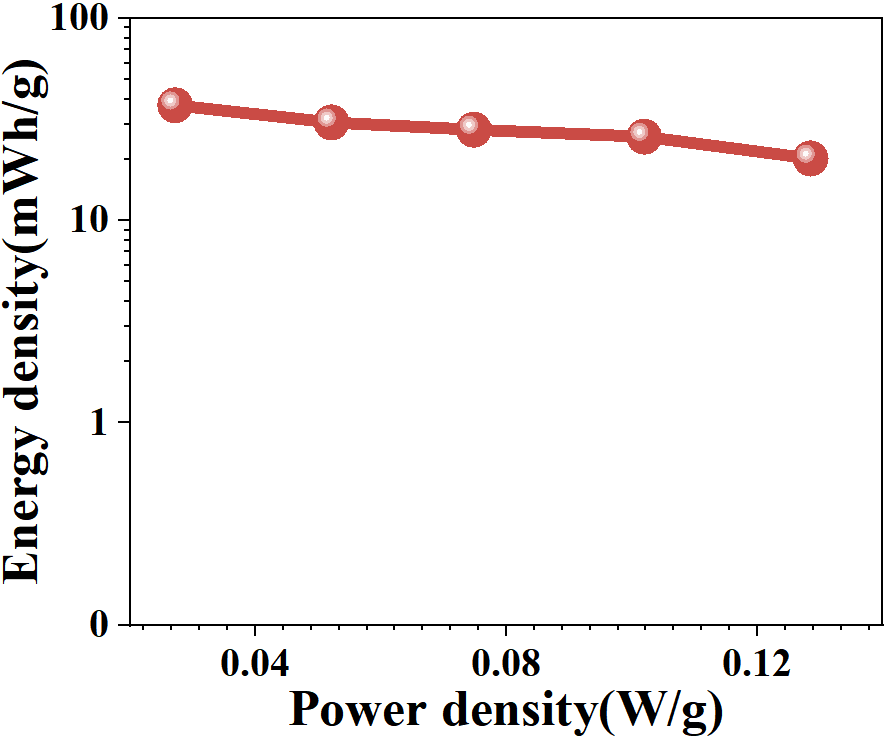


Fig. S6. Energy density and power density of Zn-Mn@FB.


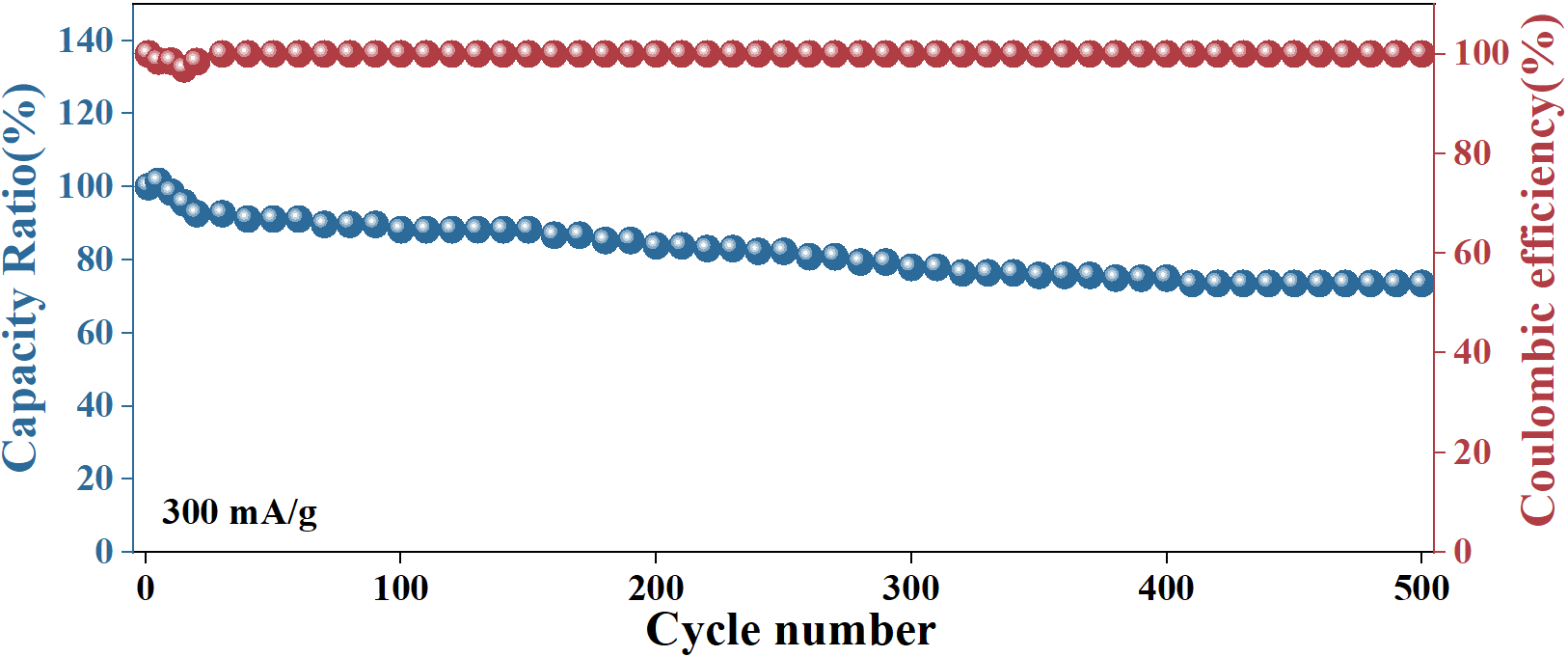


Fig. S7. Cycle stability and coulomb efficiency of the Zn-Mn@FB (500 cycles).

Fig. S8. Output current image under the soaking condition.
